# Effects of invasive plants on fire regimes and postfire vegetation diversity in an arid ecosystem

**DOI:** 10.1002/ece3.5650

**Published:** 2019-11-04

**Authors:** Emma C. Underwood, Robert C. Klinger, Matthew L. Brooks

**Affiliations:** ^1^ Department of Environmental Science and Policy University of California Davis CA USA; ^2^ Centre for Biological Sciences University of Southampton Southampton UK; ^3^ USGS Western Ecological Research Center Oakhurst CA USA

**Keywords:** *Bromus rubens*, *Bromus tectorum*, co‐occurring invasives, *Erodium cicutarium*, fire disturbance, fire frequency, Mojave Desert, *Schismus* spp.

## Abstract

We assessed the impacts of co‐occurring invasive plant species on fire regimes and postfire native communities in the Mojave Desert, western USA. We analyzed the distribution and co‐occurrence patterns of three invasive annual grasses (*Bromus rubens*,* Bromus tectorum*, and *Schismus* spp.) known to alter fuel conditions and community structure, and an invasive forb (*Erodium cicutarium*) which dominates postfire sites. We developed species distribution models (SDMs) for each of the four taxa and analyzed field plot data to assess the relationship between invasives and fire frequency, years postfire, and the impacts on postfire native herbaceous diversity. Most of the Mojave Desert is highly suitable for at least one of the four invasive species, and 76% of the ecoregion is predicted to have high or very high suitability for the joint occurrence of *B. rubens* and *B. tectorum* and 42% high or very high suitability for the joint occurrence of the two *Bromus* species and *E. cicutarium*. Analysis of cover from plot data indicated two or more of the species occurred in 77% of the plots, with their cover doubling with each additional species. We found invasive cover in burned plots increased for the first 20 years postfire and recorded two to five times more cover in burned than unburned plots. Analysis also indicated that native species diversity and evenness as negatively associated with higher levels of relative cover of the four invasive taxa. Our findings revealed overlapping distributions of the four invasives; a strong relationship between the invasives and fire frequency; and significant negative impacts of invasives on native herbaceous diversity in the Mojave. This suggests predicting the distributions of co‐occurring invasive species, especially transformer species, will provide a better understanding of where native‐dominated communities are most vulnerable to transformations following fire or other disturbances.

## Graphical abstract

This study analyzed the distribution and co‐occurrence patterns of three invasive annual grasses (*Bromus rubens*, *Bromus tectorum*, and *Schismus* spp.) known to alter fuel conditions and community structure and an invasive forb (*Erodium cicutarium*) that can dominate postfire sites in the Mojave Desert, USA. Species distribution models found most of the Mojave Desert is highly suitable for at least one of the four invasive species with up to 76% overlap between species. Analysis of 807 field plots showed that invasive cover doubled with each additional invasive taxon in a plot, invasive cover was two to five times greater where single or multiple fires had occurred, and higher levels of invasive cover were negatively associated with native species diversity. These findings provide a better understanding of where native‐dominated communities are most vulnerable to transformations following fire or other disturbances.

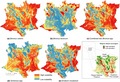

## 1 INTRODUCTION

Species that alter disturbance regimes, e.g., promoting or suppressing fire, can drive of transformation of community structure and composition (D'Antonio & Vitousek, 1992; MacDougall & Turkington, 2005; Vitousek, 1990). Many studies have shown that single invading species can impact fire frequency, fire intensity, or length of the fire season (Brooks et al., 2004). For example, invasive grasses can cause temporal changes to fire regimes causing fires to occur earlier in the season (Keeley, 2000). Moreover, modified fire regimes can facilitate an increase in distribution and/or abundance of other fire‐following invasive species which, in turn, can shorten fire‐return intervals (Lambert, D'Antonio, & Dudley, 2010).

Desert shrublands are among the most widely cited examples of invasive plants altering fire regimes through a feedback process called the grass/fire cycle (Brooks et al., 2004; D'Antonio & Vitousek, 1992). Invasive plant species richness tends to be low in deserts (Rejmánek & Richardson, 1994), but some species can be very abundant. High levels of cover of invasive annual grass (invasive grass from hereon) can increase the chance of fire ignition and facilitate fire spread, often decreasing the time interval between previous and subsequent fires as well as increasing the extent of burning (Brooks et al., 2004).

From an ecological and management perspective, this is particularly worrisome where co‐occurring invasive species exploit a similar range of environmental conditions, even if the species have different optima (i.e., peaks in abundance) along environmental gradients (species sorting; Soininen, 2014). In addition, intensity of competition from co‐occurring invasive species could exceed that of a single species and alter the structure of postfire communities over large spatial and temporal scales (Klinger & Brooks, 2017). Native desert shrubs and trees have low resilience to frequent fire, whereas annual grasses and forbs (especially non‐natives) tend to be disturbance adapted and thus have higher resilience (Brooks, 2008; Keeley, 2000). As a consequence, where fire is more frequent the abundance of woody plants decreases, while annual grasses and forbs increases. If this feedback between increasing invasive cover and increased fire frequency perpetuates, fire regimes and postfire vegetation dynamics can be severely altered (Klinger & Brooks, 2017).

In the Mojave Desert of North America, there is evidence that both the number of fires and total area burned have increased over the last several decades, and invasive annual grasses are widely regarded to be the most important causative factor (Brooks & Matchett, 2006; Brooks, Minnich, & Matchett, 2018; McKinley, Brooks, & Klinger, 2014). Three widespread invasive annual grasses, red brome (*Bromus rubens*), cheatgrass (*Bromus tectorum*), and Mediterranean grass (*Schismus* spp.: *Schismus arabicus* and *Schismus barbatus*), are known to alter fuel conditions and fire regimes (Brooks, 1999; Brooks et al., 2018) and can have long‐term effects on postfire plant communities (Klinger & Brooks, 2017). In addition, a widespread invasive forb, red stemmed filaree (*Erodium cicutarium*), often co‐occurs with these grasses and can dominate postfire landscapes due to phenological and physiological characteristics that result in strong competitive advantages over native species (Kimball, Angert, Huxman, & Venable, 2011; Kimball et al., 2014).

These four invasive taxa have been present in the Mojave Desert since the nineteenth century and are widespread and abundant, sometimes comprising as much as 99% of the annual plant community biomass (Brooks & Berry, 2006). Their relative abundance varies with elevation, with *B*. *tectorum* and *Schismus* spp. reaching their greatest abundance at upper and lower elevations, respectively, while *B*. *rubens* and *E*. *cicutarium* reach their greatest abundance at middle elevations (Brooks & Berry, 2006). This suggests habitat filtering by climate and topography, but the species are also known to co‐occur (Brooks et al., 2016; Tagestad, Brooks, Cullinan, Downs, & McKinley, 2016).

The degree of overlap in the distributions of these four invasive taxa in the Mojave Desert and the factors shaping them is largely unknown. Moreover, no studies have linked the overlapping distributions with impacts on the ground, especially in postfire vegetation communities. Understanding these relationships and impacts would allow resource managers to better predict patterns of future wildfire, anticipate postfire impacts on vegetation communities, and prioritize activities to reduce threats to ecosystem processes and native species (Brooks & Esque, 2002; Brooks & Klinger, 2009).

In this study, we determined co‐occurrence patterns between invasives and evaluated the impacts of four invasive taxa in the Mojave Desert. We addressed three questions: (a) What is the potential distribution of *B*. *rubens*, *B*. *tectorum*, *Schismus* spp., and *E*. *cicutarium* in the Mojave Desert, and how much do they overlap?; (b) What is the relationship between the abundance of invasive species and fire regimes?; and (c) Does increasing cover of invasives reduce native herbaceous diversity and evenness?

We used a two‐step approach involving species distribution modeling and analysis of field data. First, we developed species distribution models (SDMs) for the four invasive taxa to determine their potential distribution and evaluated the relationship between predicted habitat suitability and fire frequency. This step provided us with an ecoregional‐scale assessment of their individual and joint distributions, what factors shaped those distributions, and the strength of the association between fire frequency and where the taxa were most likely to occur. Second, we analyzed the cover of the four invasive taxa from field plots and fire history (fire frequencies and years postfire [YPF]), and native herbaceous diversity along a gradient of invasives cover. We reasoned that (a) an increase in invasive cover as fire frequency increased would indicate a relationship between habitat suitability and fire frequency and (b) a decrease in native diversity as invasive cover increased would indicate the potential impacts suggested by the SDMs was being realized.

## 2 METHODS

The Mojave Desert is the smallest of the four major desert regions in North America (≈152,000 km^2^ in area). Topography is extremely varied, with elevation ranging from −85 to 3,635 m, though most of the ecoregion is between 600 and 1,600 m. Temperature extremes vary from −20°C (high elevation) to −5°C (low elevation) during the winter and highs of 30°C (high elevation) to 50°C (low elevation) in the summer. Precipitation in the western Mojave occurs mostly during the winter (≈82%–87%), whereas, in the eastern Mojave, precipitation is more bimodal (winter ≈71% and summer ≈29%) (Hereford, Webb, & Longpre, 2006; Tagestad et al., 2016).

### 2.1 Data collection

#### 2.1.1 Fire data

Fire perimeter data across the Mojave Desert 1984–2010 were acquired from the Monitoring Trends in Burn Severity (MTBS) database (http://www.mtbs.gov/), which provides data across the western USA on fires >1,000 acres (405 ha). Data generated from MTBS protocols has a relatively high mapping precision and consistent methodology compared to other types of fire data (Brooks, Matchett, Shinneman, & Coates, 2015; Eidenshink et al., 2007).

We extended our timeframe beyond that of the MTBS data by creating fire perimeter layers from 1972 to 1983 using similar data sources and methods as the MTBS program. Specifically, we used Landsat 4/5 Multispectral Scanner (MSS) imagery to select two or more high‐quality scenes for each Landsat path/row required to provide full study area coverage. We selected one scene during the spring (April, May, and June) and one in late summer and early fall (August, September, and October) of each year, corresponding to the beginning and end of the fire season in the Mojave Desert. We selected scenes to minimize snow and cloud cover as well as other anomalies (e.g., missing data) sometimes found in older Landsat imagery. MSS imagery lacks the shortwave infrared (SWIR) spectral band required to derive the difference normalized burn ratio (dNBR), which represents the standard index of burn severity in the MTBS program. Therefore, we incorporated images of the differenced normalized difference vegetation index (dNDVI), which has been shown to provide comparable estimates of burn severity as dNBR (Hudak et al., 2007; Zhu, Key, Ohlen, & Benson, 2006). The resolution of MSS imagery is also coarser than that of later Landsat imagery (79 m vs. 30 m, respectively); therefore, we used supplementary geospatial data (e.g., elevation, topographic maps) to assist in making final perimeter delineations. The dNDVI and additional geospatial data we used to aid in our interpretation of the imagery made differences in resolution a minor issue, and we successfully identified fire perimeters from 1972 to 1983. After assembling images from 1972 through 2010, we calculated fire frequency on a pixel by pixel basis by overlaying fire perimeters and mapping their overlap (summing from one fire to six fires) over time (1972–2010, using ArcGIS; ESRI http://www.esri.com/).

#### 2.1.2 Plant community data

We used 807 plots of vegetation data within (burned) or adjacent to (unburned) perimeters of 53 fires that overlapped (partially or entirely) with the Mojave Desert ecoregion boundary (Klinger & Brooks, 2017). We preselected randomly located vegetation plots within each fire perimeter using fire frequency and burn severity data (dNBR and dNDVI), which also helped identify potentially confounding landscape features (e.g., cliffs, unburned islands of vegetation within fire perimeters). Burned plot locations (*N* = 578) were finalized after field visits to verify direct fire evidence (e.g., charcoal, shrub, and tree stumps; Klinger & Brooks, 2017). In contrast, we finalized the locations of the unburned plots (*N* = 229) based on lack of fire evidence.

To account for variability related to topography, soils, and historical and current land use, we randomly selected 1‐km^2^ sites entirely within or outside fire perimeters, then randomly selected 3–7 plots within each site. We stratified the plots (32 m × 32 m; 0.1 ha) by intervals of years postfire (YPF; 1–5, 6–10, 11–20, and 21–40 YPF), fire frequency (burned 1–3 times since 1972 or unburned), and elevation zone (low zone < 1,200 m, mid‐zone 1,200–1,700 m, and high zone > 1,700 m; Klinger & Brooks, 2017). We avoided areas with disturbances such as mining and watering holes, although a small number of plots (<30) had been grazed historically (>40 years) by livestock. Sampling occurred in the spring (March through May) of 2009, 2011, 2012, and 2013 and timed to match peak vegetation production in each elevation zone (Table S1). We sampled each plot once during the study.

We recorded the occurrence (presence/absence) of the four invasive taxa in a series of nested quadrats (1, 10, 100, 250 and 1,000 m^2^) within each 0.1‐ha plot (Figure S1). The nested quadrats gave us an accurate method for determining if the invasives occurred in a plot but not for making meaningful estimates of their abundance in the larger quadrats (>10 m^2^). Therefore, we recorded ocular estimates of cover for all herbaceous plants (perennial, annual, and biennial for native and non‐native species) in 15 randomly located 1‐m^2^ quadrats (0.5 m × 2 m) within each plot (Figure S2). We used occurrence data to model the distribution of the four invasive taxa throughout the Mojave Desert and cover data to evaluate the effects of the four taxa on the native herbaceous community.

Given the variability associated with invasive species cover over the four years of sampling (e.g., associated with interannual differences in rainfall), we also sampled invasive taxa occurring in the seedbank. We collected seedbank samples in a randomly selected subset of the 0.1‐ha plots (*N* = 618; 179 unburned, 439 burned) from late September through mid‐October, corresponding to the year of the aboveground vegetation data collection. We collected four samples in 6 cm diameter × 3 cm deep soil tins from just outside of each 1‐m^2^ cover quadrat. The samples for each quadrat were composited and grown out in a greenhouse in Bishop, California. We recorded the number of seedlings per quadrat that emerged for each species after 9–12 months of watering treatments. The combination of aboveground and seedbank sampling provided us with occurrence data from 618 plots for each of the four invasive taxa.

### 2.2 Data analysis

#### 2.2.1 Representation of ecoregional environmental conditions

We used a principal components analysis (PCA) to evaluate the degree to which environmental conditions at our plots were representative of the ecoregion (since invasive annual plants in the Mojave Desert vary with elevation and associated temperature and precipitation; Brooks & Berry, 2006; Brooks et al., 2016). We extracted attributes of eight variables for each field plot as well as 10,000 points generated randomly across the ecoregion. The variables included elevation (30 m spatial resolution), slope (30 m), aspect (cosine transformed, 30 m), and mean minimum January temperature (1950–2005), mean maximum July temperature (1950–2005), mean wet (November–April), and dry season (May–October) precipitation (1950–2005) from climate data with a native resolution of 860 m (ClimSurf; Alvarez, Guo, Klinger, Li, & Doherty, 2013), and mean peak normalized difference vegetation index (NDVI, 2000–2010, 250 m native resolution; seasons based on Tagestad et al., 2016). We used the correlation matrix of the eight variables for the PCA and the Kaiser‐Guttman criterion for retention of PCA axes (McGarigal, Cushman, & Stafford, 2000).

#### 2.2.2 Species distribution modeling

We derived variables for the SDMs from the 618 plots with aboveground and seedbank data for the four invasive taxa. Variables included (a) topographic data: elevation, slope, aspect (eight classes), and potential solar radiation; (b) climate data: total annual precipitation and total precipitation for summer, spring, and winter seasons (means calculated from 1950 to 2005), mean annual temperature, and mean maximum and minimum temperatures from 1950 to 2005 (Alvarez et al., 2013), potential evapotranspiration (PET), actual evapotranspiration (AET), surplus and deficit (derived using the AET Calculator v.1.0; http://geog.uoregon.edu/envchange/pbl/software/AETcalculator.pdf); and (c) vegetation‐related data: vegetation class (Multi‐Resolution Land Class dataset), percent tree cover, percent herbaceous cover, and percent bare ground derived from MODIS data (DiMiceli et al., 2011), peak NDVI, and standard deviation of NDVI. We resampled spatial data to 30‐m resolution where necessary.

We predicted habitat suitability across the ecoregion for each invasive species using presence and absence data in a generalized linear model (GLM) with a binomial error structure and logit link. We developed models for *E. cicutarium*, *Schismus* spp. (*S. barbatus* and *S. arabicus*), *B. rubens*, and *B. tectorum*, as well as a model for the two *Bromus* species combined. We combined data for the two *Bromus* species based on their high rate of co‐occurrence (35% of plots where at least one of the four invasive taxa occurred [*N* = 787] contained both *B. rubens* and *B. tectorum*), their similar response to fire, and their mutual role as fire promoting species. Data were combined for *S. barbatus* and *S. arabicus* because of difficulties in reliably distinguishing them in the field. To develop the GLMs, we used data from both burned and unburned plots (71% of the 618 plots had burned since 1972; Klinger & Brooks, 2017; R. McKinley, USGS EROS, unpublished data). To avoid circularity, we did not include variables relating to fire history, as our objective aimed to evaluate physiographic influences on plant distributions and not disturbance from fire.

We grouped input variables into topography, climate, and vegetation sets and then assessed Pearson pairwise correlations among the variables within each set (intra‐set correlations). Where pairwise correlations were >0.70, we selected one of the variables from each set to include in the GLM (Table S2). After removing redundant variables within sets, we repeated the procedure for the remaining variables across sets (intra‐set correlations). We evaluated model fit with a Chi‐square test and validated the models using 50 replicates of a 10‐fold and leave‐one‐out cross‐validation. We tested the strength of the variables by sequentially dropping second‐order, then first‐order variables and used AICc to evaluate whether the model improved without the variable (Zuur, Leno, Walker, Saveliev, & Smith, 2009), until ΔAICc > 2. We used the significant variables from the best GLM to create a predicted suitability layer ranging from 0 (least suitable) to 1 (highly suitable) for each invasive species and the combined *Bromus* spp. (glm function in the base distribution package of R, R Core Team, 2014).

To confirm the level of co‐occurrence between *B. rubens*, *B. tectorum*,* E. cicutarium*, and *Schismus* spp. (*S. barbatus* and *S. arabicus*), we generated a co‐occurrence matrix based on the above‐ground cover data in plots where at least one of the four species occurred (Table S3). To evaluate the potential impacts from the four taxa, we used the coefficients from their respective GLMs to predict probability of occurrence along gradients of mean annual temperature, mean total annual precipitation, and mean peak NDVI, variables that significantly contributed to the models for all four taxa. We plotted the curves for each species and visually inspected their range, peak level (optima), and rate of change along each of the three gradients.

Finally, we assessed a worst‐case scenario for resource managers of the spatial co‐occurrence of the two *Bromus* species (which provide fuel) and *E. cicutarium* (which impacts postfire recovery) by assigning the predicted suitability values of each species to four classes for comparison (very high, high, medium, and low) using Jenk's natural breaks (Table S4). We identified the spatial overlap of areas classified as very high for both species and also low for both species.

#### 2.2.3 Relationship between invasive species and fire frequency

We analyzed predicted habitat suitability patterns of the invasive taxa in relation to spatial patterns of fire frequency including single burns (one fire occurrence 1972–2010), multiple burns (between 2 and 6 burns occurring 1972–2010), and unburned areas (see Section 2.1). We generated 10,000 random points within the perimeters of single burns, 10,000 random points within the perimeters of multiple burns, and 10,000 random points outside of burn perimeters. We extracted the predicted suitability values at each point and generated kernel density curves for unburned, single‐burn, and multiple‐burn classes. We then used bootstrap sampling (*N* = 100 samples, with 1,000 random points (10% of total) per bootstrap sample to calculate means and 95% confidence intervals) of the predicted suitability values in the unburned, single‐burn, and multiple‐burn classes.

#### 2.2.4 Effects of the four invasive taxa on herbaceous vegetation

We calculated the number of invasive and native species and mean individual and cumulative cover per plot of *B. rubens*, *B*. *tectorum*, *Schismus* spp., and *E*. *cicutarium* from the herbaceous cover data. We also calculated three indices to reflect native species diversity (Hill, 1973): *N*_0_ (species richness); *N*_1_ (exp^H′^, where H′ is Shannon's index); and *N*_2_ (1/*S*, where *S* = Simpson's index). These indices (members of Hill's series of diversity indices) are particularly informative as the values are interpreted as effective numbers of native species, making it one of the most useful measures of diversity (Jost, 2006; Routledge, 1979). We selected *N*_0_, *N*_1_, and *N*_2_ because they span a useful range in sensitivity to rare species, with *N*_0_ being the most sensitive and *N*_2_ the least. We also calculated Pielou's index of evenness for each plot (Jost, 2010):

$$J=\frac{H^{\prime }}{\ln(N_{0})}$$

These four indices allowed us to assess how changes in native diversity were related to increasing cover of the invasive taxa and whether changes were an outcome of decreased richness, evenness, or both. For example, a significant decline in *N*_0_ with increasing cover of the invasive taxon would indicate the local loss of native species; alternatively, a minimal change in *N*_1_, *N*_2_, and *J* would suggest less common native species were being displaced.

#### 2.2.5 Data analyses

We used generalized linear mixed models (GLMMs) to analyze (a) the relationship between the total number of the four invasive species present in a plot and their cumulative absolute cover (logit transformed) in a plot; (b) the relationship between total absolute and relative logit transformed cover of the four invasive species (i.e., summed across species) with YPF and fire frequency; and (c) the relationship between the diversity and evenness indices with cumulative relative cover of the invasive taxa.

To analyze the total number and cover of the four invasive species, we specified the number of species as a fixed effect and site as a random effect. We compared random slope + intercept and random intercept‐only models with the bias‐corrected version of the Akaike information criterion (AICc) and AICc weights (*w*AICc) (Burnham & Anderson, 2002). To analyze the relationships between total absolute and relative cover of the invasives with YPF and fire frequency, we specified five models that we compared with AICc and *w*AICc. These included the additive effects of fire frequency and YPF^2^, the additive effects of fire frequency and YPF, fire frequency, YPF, and a null model. Fixed effects included YPF and fire frequency, and the random effects included site. We did not include year of sampling as a random effect because there were too few levels (*N* = 4) (Bolker et al., 2009), and a preliminary analysis indicated site accounted for 5×–10× more of the model deviance than year of sampling.

To analyze diversity and evenness indices, we used relative cover of the invasive taxon as the fixed effect and site the random effect in the models. However, we conducted a preliminary analysis where we used AICc and *w*AICc to compare random slope + intercept and random intercept‐only models. After determining the most appropriate random effects structure (Zuur et al., 2009), we analyzed three fixed effect models: the nonlinear effect of invasive relative cover, the linear effect of invasive relative cover, and a null model. We used a Poisson error structure and log link for the models with *N*_0_ and a Gaussian error structure and identity link for models with *N*_1_, *N*_2_, and logit transformed *J*.

## 3 RESULTS

### 3.1 Potential distribution of invasive taxa in the Mojave Desert

**Figure 1:**
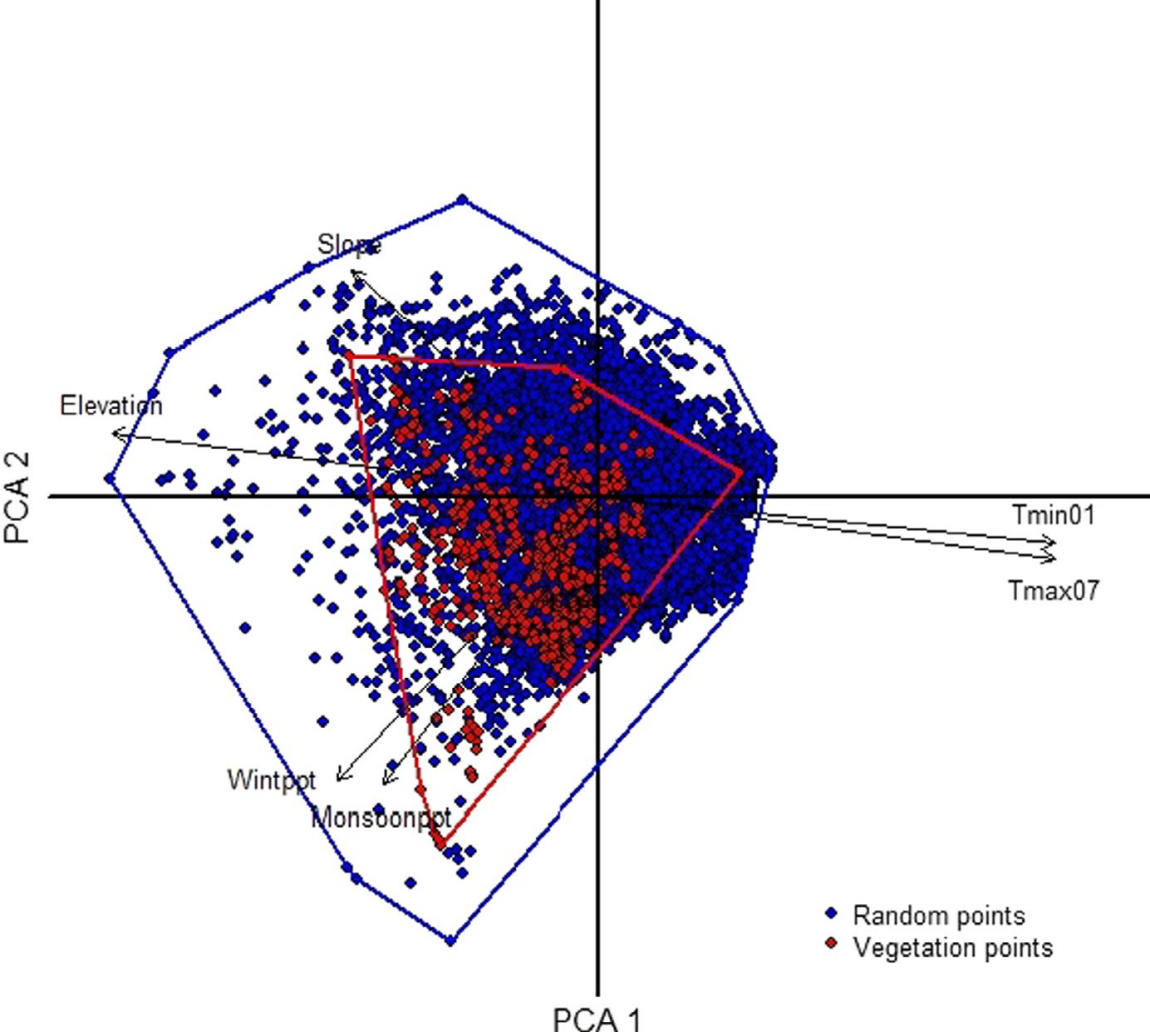
PCA ordination showing the surveyed vegetation plots (red dots) with environmental variables. The ordination indicates adequate representation of the environmental envelop of the Mojave Desert ecoregion (Wintppt = winter precipitation, Monsoonppt = monsoon (summer) precipitation, tmin01 = minimum temperature, tmax07 = maximum temperature, along with slope and elevation)

We first assessed plots to ensure they captured a range of environmental variables. The PCA indicated field plots adequately represented the range of environmental conditions in the ecoregion, though there was a tendency for plots to not be in the very lowest and highest elevations of the region (Figure 1). Three components had eigenvalues >1 and cumulatively explained 72.2% of the variance in the data. A gradient in temperature explained the first component (44.1% of the variance), a gradient in precipitation and slope and explained 15.5% of the variance, and an aspect gradient (primarily) explained the third component (12.6% of the variance).

**Figure 2:**
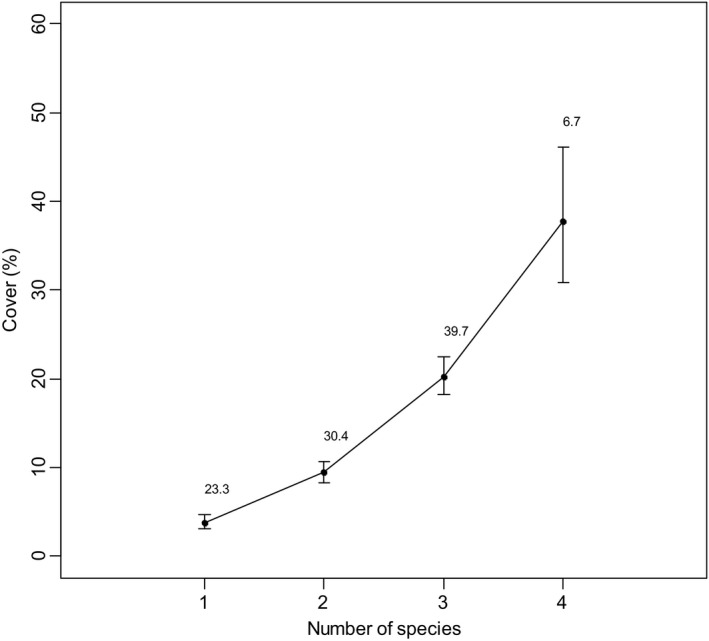
Mean percent absolute cover (±95% CIs) of the invasive taxa in 0.1‐ha plots (*N* = 718) in the Mojave Desert where an invasive taxa *Bromus rubens*,* Bromus tectorum*, *Schismus* spp. (*S. arabicus* and *S. barbatus*), and *Erodium cicutarium* occurred either singly or in combination with one of the other species. Numbers above the upper CI are the percentage of plots when the species occurred singly or in combination with one of the other species. The plots were sampled in 2009, 2011, 2012, and 2013

Analyses showed at least one of the four invasive taxa occurred in 91% of the plots with herbaceous cover, and of those plots (*N* = 718), two or more of the species co‐occurred in 77% (Figure 2). We found a consistent increase in invasive cover as the number of invasive taxa in a plot increased (Figure 2). Invasive cover essentially doubled with each additional invasive species in a plot, with 8× greater invasive cover in plots with four of the taxa compared to one.

### 3.2 Species distribution modeling

The 618 plots provided a solid representation of the occurrence and co‐occurrence of the four invasive taxa (Table S3). Analysis showed at least one of the four taxa was present in 585 of the aboveground samples (94.6%), while at least one of the four taxa was present in 357 of the seedbank samples (57.8%). There were only five seedbank samples where at least one of the four taxa was present but not present in the aboveground samples (<1%). There were 28 plots (4.5%) where the four taxa were not present in the aboveground or seedbank samples. *Bromus rubens* occurred in 541 plots, *E. cicutarium* in 474 plots, *B. tectorum* in 372 plots, and *Schismus* spp. in 210 plots.

We dropped thirteen of the 20 predictor variables after inspecting the pairwise correlations. The remaining seven variables (*r* < 0.53) in the GLMs included slope, aspect, solar radiation, mean temperature, total annual precipitation, peak NDVI, and percent tree cover, as well as the second‐order (nonlinear) effects of mean temperature, total annual precipitation, and NDVI (Table 1). Mean temperature had strong predictive value for all four species, and mean annual precipitation and peak NDVI had strong predictive value for all but one species each (Table 1). The GLMs for each species performed well, with consistency across the minimum, median, mean, and maximum values in the 10‐fold and leave‐one‐out cross‐validation tests generally indicating high model accuracy. Root mean square error values ranged from a low of 1.30% (*SE* 0.056) for *B. tectorum* to 4.76% (*SE* 0.138) for *E. cicutarium*. *Erodium cicutarium* had the highest pseudo *r* ^2^ of the models (*r* ^2^ = 0.61) and *B. tectorum* the lowest (*r* ^2^ = 0.19) (Table 1), with values above 0.2 indicative of excellent fit (McFadden, 1974).

**Table 1:** Key variables for predicting the suitability of habitat in the Mojave Desert ecoregion, identified in the GLM

	2 *Bromus* combined			*Bromus rubens*			*Bromus tectorum*			*Schismus* spp.			*Erodium cicutarium*		
	Estimate	*SE*	*p*	Estimate	*SE*	*p*	Estimate	*SE*	*p*	Estimate	*SE*	*p*	Estimate	*SE*	*p*
(Intercept)	−6.991	1.217	<.0001	−6497	1,580	<.0001	93.453	11.709	<.0001	−229.800	20.600	<2e−16	−14,480	2,603	<.0001
Annual PPT	0.013	0.006	.026	0.015	0.005	.001	0.016	0.002	<.0001	–	–	–	0.030	0.012	.009
Annual PPT^2^	–	–	–	–	–	–	–	–	–	–	–	–	0.000	0.000	.023
Peak NDVI	0.004	0.001	<.0001	0.002	0.001	<.0001	–	–	–	0.003	0.001	<.0001	0.003	0.000	<.0001
Peak NDVI^2^	0.000	0.000	<.0001	0.000	0.000	<.0001	–	–	–	0.000	0.000	<.0001	–	–	–
% Tree cover	−0.394	0.110	<.0001	–	–	–	–	–	–	–	–	–	−0.870	0.160	<.0001
Mean Temp	–	–	–	44.960	11.030	<.0001	−0.335	0.041	<.0001	0.780	0.071	<.0001	100.200	18.100	<.0001
Mean Temp^2^	–	–	–	−0.078	0.019	<.0001							−0.173	0.031	<.0001
Solar Rad.	–	–	–	–	–	–	–	–	–	–	–	–	0.003	0.001	.041
	Pseudo *r* ^2^ =	0.339		Pseudo *r* ^2^ =	0.368		Pseudo *r* ^2^ =	0.198		Pseudo *r* ^2^ =	0.311		Pseudo *r* ^2^ =	0.611	
	Rmspe	3.441		rmspe	2.539		rmspe	1.300		rmspe	2.503		rmspe	4.761	
	*SE*	0.059		*SE*	0.062		*SE*	0.056		*SE*	0.082		*SE*	0.138	

Abbreviations: Annual PPT, annual precipitation; Mean Temp, mean temperature; Peak NDVI, peak normalized difference vegetation index; Solar Rad., solar radiation.

**Figure 3:**
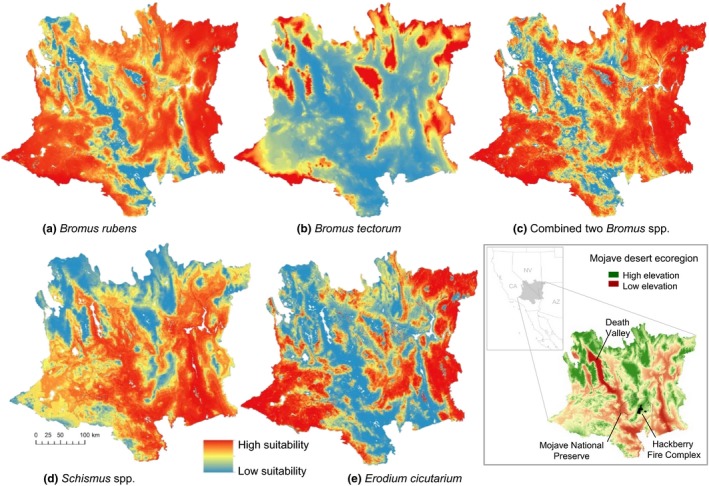
Locator map and predicted ecoregion suitability for five invasive plants based on a GLM using presence/absence data from 618 plots in the Mojave Desert ecoregion: (a) *Bromus rubens*, (b) *Bromus tectorum*, (c) combined two *Bromus* spp. (d) *Schismus* spp., and (e) *Erodium cicutarium*

**Figure 4:**
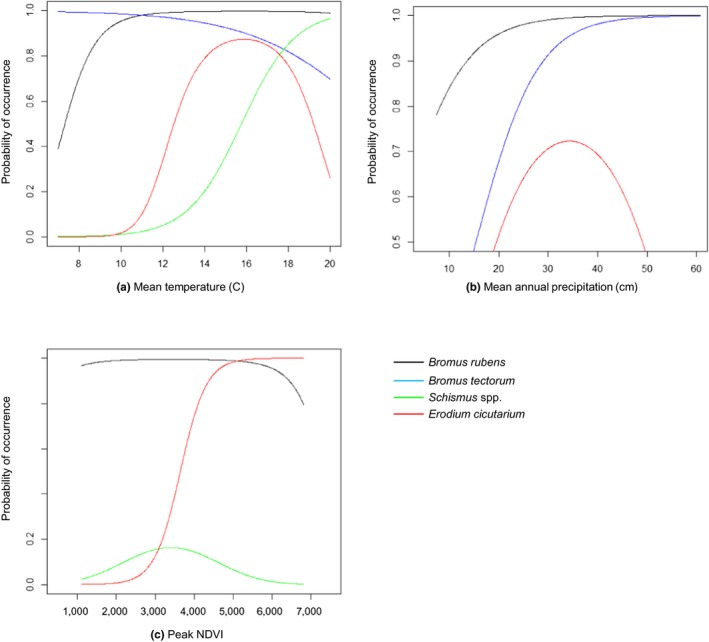
Probability of occurrence of four invasive plants in response to the three most frequent environmental predictors in the Mojave Desert ecoregion: (a) mean temperature, (b) mean total annual precipitation, and (c) peak NDVI. Dotted lines represent ± 95% confidence intervals

Predicted suitability patterns varied across the ecoregion for each of the four invasive taxa. *Bromus tectorum* and *E. cicutarium* co‐occurred in 47.4% of the 618 plots and *B. rubens* and *E. cicutarium* co‐occurred in 74.1%, resulting in similar suitability maps (Figure 3a,e). Mean temperature and annual precipitation significantly predicted the probability of occurrence for both species, although *B. rubens* showed a rapid increase and plateau response to increasing temperature and precipitation, while *E. cicutarium* had a bell‐shaped response resulting in a lower proportion of the ecoregion being highly suitable (Figure 4a,b). Correspondingly, the SDM showed much of the Mojave Desert suitable for *B. rubens*, except in the very coolest (mountainous) and driest (low lying) areas (Figure 3a). In contrast, the SDM for *Schismus* indicated high suitability in areas with warmer temperatures, such as Death Valley (Figure 2d and locator map, Figure 4a).

**Figure 5:**
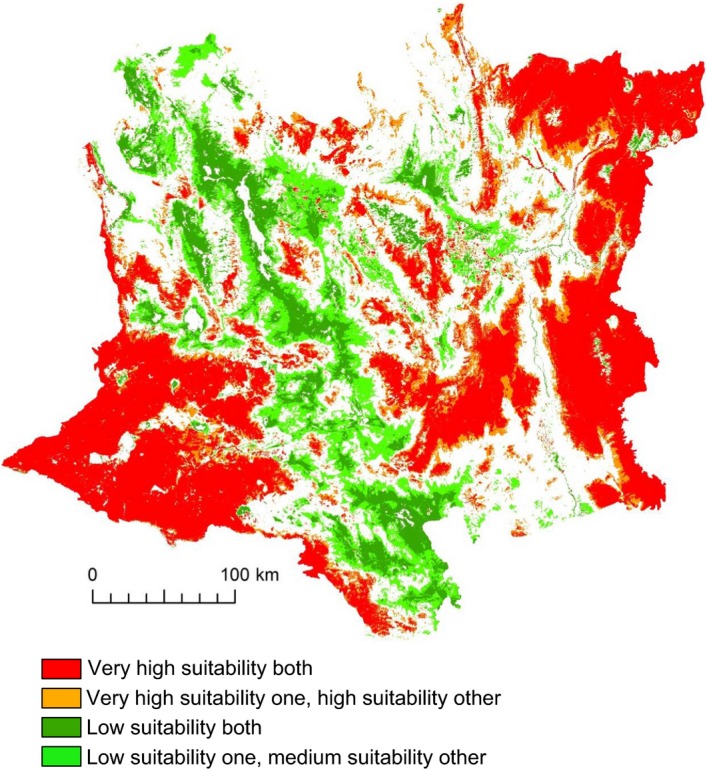
Spatial pattern of the highest and lowest predicted suitability classes across the Mojave Desert ecoregion when *Bromus* spp. (*B. rubens* and *B*. *tectorum* combined) or *Erodium cicutarium* occurred singly (“one”), or when *Bromus* spp. and *E. cicutarium* occurred in combination (“both”)

Outputs from the SDMs predicted much of the ecoregion was highly suitable for one or more of three invasive grasses and *E. cicutarium* (Figure 5). We classified 76% (9.8 million ha) of the Mojave Desert as high or very high suitability for both *B*. *rubens* and *B*. *tectorum*. However, 42% (5.3 million ha) of the Mojave Desert had high or very high suitability for the overlapping distributions of *Bromus* spp. (which alters fuel conditions) and *E. cicutarium* (which hinders postfire native revegetation) (Figure 5, Table 2). In contrast, only 8% (1 million ha) of the landscape had low predicted suitability for both the *Bromus* spp. and *E. cicutarium* (Table 2).

**Table 2:** Summary (ha and proportion [%]) of the spatial overlap between the predicted suitability for two *Bromus* spp. and *Erodium cicutarium* in the Mojave Desert ecoregion

	ha	*E. cicutarium*				
		Low	Medium	High	Very high	Total
2 *Bromus* spp.	Low	1,090,306 (8%)	36,428 (0%)	3,668 (0%)	91,566 (1%)	1,221,969 (9%)
	Med	1,471,465 (11%)	456,527 (4%)	2,446 (0%)	4,222 (0%)	1,934,660 (15%)
	High	1,151,175 (8%)	1,218,739 (9%)	717,656 (6%)	10,233 (0%)	3,097,804 (24%)
	Very high	718,362 (6%)	571,142 (4%)	1,231,494 (10%)	4,149,494 (32%)	6,670,493 (52%)
	Total	4,431,309 (34%)	2,282,837 (18%)	1,955,265 (15%)	4,255,515 (33%)	12,924,926

### 3.3 Relationship of invasive species and fire frequency

**Figure 6:**
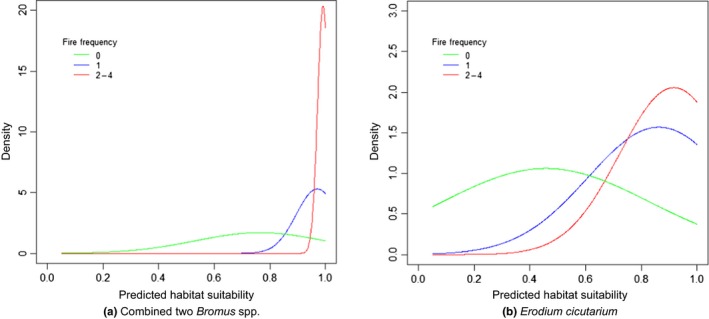
Differences between the predicted suitability values for invasives across different fire frequency classes (single burn, multiple, burns and unburned) in the Mojave Desert ecoregion. Results show 100 bootstrap samples (*n* = 1,000 per class) distribution and fire frequency means for (a) the combined two *Bromus* spp. predicted suitability model and (b) *Erodium cicutarium* model

The predicted suitability varied between unburned, single‐burn, and multiple‐burn areas (Table 3, Figure 6) and, with one exception, we found a clear relationship between invasion suitability and fire frequency. For the two invasive grasses (*B. rubens* and *B. tectorum*) combined (Figure 6a) and *E. cicutarium* (Figure 6b), the highest mean invasion suitability value occurred in areas with multiple fires and lowest in unburned areas. Bootstrap resampling of mean suitability values for multiple burns ranged from a high of 0.991 for the combined *Bromus* spp. model to 0.713 for *B. tectorum* alone (Table 3, Figure 6a). Lowest suitability values for unburned areas ranged from a high of 0.765 for the two *Bromus* spp. combined to 0.274 for *B. tectorum* alone (Table 3). In contrast to the patterns above, *Schismus* spp. had the highest mean suitability in unburned areas (0.581, Table 3) and lowest suitability in areas of multiple fires (0.317).

**Table 3:** Mean and standard error from 100 bootstrap samples (*n* = 1,000 per class) of the predicted invasion suitability layers and three classes of fire frequency (single burn, multiple burns, and unburned) in the Mojave Desert ecoregion. Bold indicates highest mean value for each model

	2 *Bromus* spp.		*B. rubens*		*B. tectorum*		*Schismus* spp.		*E. cicutarium*	
	Mean	*SE*	Mean	*SE*	Mean	*SE*	Mean	*SE*	Mean	*SE*
Unburned	0.7648	0.0006	0.7374	0.0007	0.2736	0.0007	**0.5811**	0.0009	0.4567	0.0012
Single fire	0.9707	0.0002	0.9261	0.0004	0.6342	0.0007	0.3582	0.0008	0.8641	0.0008
Multiple fires	**0.9916**	0.0001	**0.9545**	0.0003	**0.7132**	0.0007	0.3174	0.0008	**0.9165**	0.0006

We found complete support for the model with the additive effects of fire frequency and YPF^2^ on absolute cover of the invasive taxa (*w*AICc = 1; Table S5). We found absolute cover of the invasive taxa greatest between 10 and 20 YPF (Table 4): it was 2× to almost 3× greater in plots that had burned once and were < 20 YPF than in unburned plots. However, in plots that had burned more than once and were <20 YPF, it was 3×–5× that in unburned plots.

**Table 4:** The mean (±95% CIs) absolute and relative cumulative cover of the invasive taxa *Bromus rubens*, *Bromus tectorum*, *Schismus* spp. (*S. arabicus* and *S. barbatus*), and *Erodium cicutarium* in three fire frequency and five years postfire classes in the Mojave Desert. There were 807 plots (0.1 ha) that were sampled once in either 2009, 2011, 2012, or 2013. Fire frequency represents the number of fires since 1974 (0 = unburned)

Frequency	Years postfire	Estimate	95% CI
Absolute cover (%)			
Unburned	–	4.7	4.3–5.1
Burned once	1–5	10.5	9.9–11.1
	6–10	12.6	11.8–13.6
	11–20	14.2	12.8–15.7
	21–40	6.1	5.4–7.0
Burned two or more times	1–5	17.0	15.4–18.7
	6–10	20.2	18.6–22.0
	11–20	22.4	20.5–24.6
	21–40	10.3	8.9–11.9
Relative cover (%)			
Unburned	–	23.2	20.1–26.6
Burned once	1–5	50.1	47.0–53.2
	6–10	56.0	52.0–59.9
	11–20	60.9	55.2–66.2
	21–40	43.6	37.4–50.1
Burned two or more times	1–5	70.6	65.7–75.0
	6–10	75.3	71.2–78.9
	11–20	78.8	74.7–82.4
	21–40	64.9	57.6–71.6

Absolute cover of the invasive taxa decreased between 20 and 40 YPF but did not reach unburned levels; cover in plots that burned once was 1.5× that in unburned plots, while cover in plots that had burned two or more times was more than 2× that in unburned plots, even after almost four decades (Table 4).

The model with the additive effects of fire frequency and YPF^2^ on relative cover of the invasive taxa had more than 2× the support as a model with just fire frequency (Table S5). Relative cover of the invasive taxa showed a similar pattern as absolute cover (Table 4). They comprised the dominant herbaceous cover (>50% relative cover) across all YPF classes in plots that burned two or more times, and the dominant herbaceous cover the first 20 YPF in plots that had burned once. Although their relative cover decreased between 20 and 40 YPF, it remained nearly 2× greater in plots that had burned once and nearly 3× greater in plots that burned more than once than in unburned plots (Table 4).

### 3.4 Effects of the four invasive taxa on herbaceous vegetation

**Figure 7:**
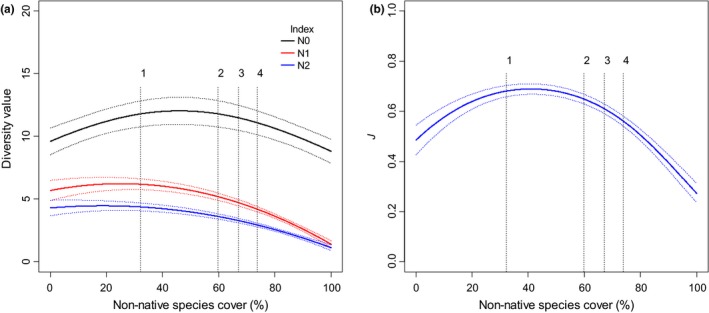
The relationship (±95% CIs) that three diversity indices (Hill's series *N*_0_, *N*_1_, and *N*_2_; a) and an index of evenness (Pielou's *J*; b) of herbaceous plants had with cumulative relative cover of the invasive taxa *Bromus rubens*,* Bromus tectorum*, *Schismus* spp. (*S. arabicus* and *S. barbatus*), and *Erodium cicutarium* in 787 plots (0.1 ha) sampled once in either 2009, 2011, 2012, and 2013 in the Mojave Desert. The dashed vertical lines are the mean values of relative cover when 1, 2, 3, or 4 of the invasive taxa were present (number of taxa are shown above the dashed lines)

We determined complete support for models with a quadratic relationship between the diversity and evenness indices and invasive relative cover (Table S5). Analysis showed no substantial evidence that native herbaceous species were lost as invasive relative cover increased; *N*_0_ reached its maximum values in the intermediate range of invasive relative cover, and while it decreased beyond that, the values of *N*_0_ at the higher end of the range of invasive relative cover were very similar to those in the lower end of the range (Figure 7a). In contrast, values for *N*_1_ and *N*_2_ were relatively constant up to approximately 40% relative cover of the invasive taxon. Beyond that, they decreased, and at the higher end of the range, invasive relative cover comprised less than half that at the lower end (Figure 7a). *J* peaked in the intermediate range of invasive relative cover, but then decreased rapidly to levels less than half of those at the lower end of the range (Figure 7b). Collectively, these patterns indicated the lower levels of native herbaceous diversity at high levels of invasive relative cover were a result of a decline in abundance of more common species.

## 4 DISCUSSION

Our combination of ecoregional‐scale models and analyses of an extensive set of plot data showed the effects of four invasive taxa on fire frequency and native herbaceous diversity in postfire communities. Predicting invasive species distributions using SDMS constituted an important component of this project, but there can be limitations in using them. Detectability of species is often a distinct challenge (Fielding & Bell, 1997), but we minimized this by (a) sampling a relatively large number of quadrats within each plot; (b) systematically inventorying all species in each plot; (c) sampling across multiple years (e.g., with different precipitation); and (d) sampling the soil seedbank to minimize chances we erroneously recorded one of the species as absent when it was not observed aboveground. Consequently, we believe that the absences we recorded were true absences. Another potential limitation is how representative samples are of environmental conditions (Peterson et al., 2011), but the PCA (Figure 1) indicated that the distribution of our plots adequately sampled the major topographic and climatic gradients in the ecoregion. The SDMs indicated very high suitability of most of the region for *B. rubens*, *B. tectorum*, *Schismus* spp., and *E*. *cicutarium*, with a large proportion predicted to be highly suitable for co‐occurrence of two or more of the taxa (Figure 5). For example, we estimated overlap of areas with predicted high and very high suitability for both *Bromus* spp. and *E. cicutarium* to encompass 42% of the region. The plot data supported the high degree of co‐occurrence predicted by the SDMs, with *B. rubens* co‐occurring with *E. cicutarium* in over half of the plots (56%) and *B. tectorum* and *E. cicutarium* co‐occurring in one‐third of the plots. The analysis of plot data indicated that invasive cover essentially doubled with each additional invasive taxon in a plot.

The invasive species we assessed are known to promote recurrent fire and/or reduce resilience in postfire vegetation communities in the North American deserts, so an increase in their cover greatly increases the potential for long‐term change in postfire communities and fire regimes (Brooks & Chambers, 2011; Brooks et al., 2018). Our findings indicate this potential is likely being translated into actual impacts. First, the relationship between fire frequency and predicted SDM values for the annual grasses pointed to the well‐documented feedback between annual grasses and short fire return intervals (Brooks et al., 2004). Second, we recorded two to five times more invasive cover in the plot data in single‐ or multiple‐burned plots than in unburned plots. In addition, we found invasive cover a significant or dominant portion of the community for at least 20 years following fire. Third, the abundance of the more common native herbaceous species declined when invasive relative cover >60%–70%, resulting in a decrease in several indices of diversity. This nonlinear relationship likely reflected gradients in habitat conditions, resources, biotic interactions, or a combination of the three. For example, some site conditions were probably not sufficiently suitable for high abundance of the invasive taxa, or resource levels were high enough and/or competition not intense enough for invasive taxa to suppress cover of native herbaceous species (Maestre, Callaway, Valladares, & Lortie, 2009; Riesch, Plath, & Bierbach, 2018). However, at a certain point invasive cover does reach a level where it begins to reduce abundance of native herbaceous species, which in our study appeared to be the more common ones (indicated by the declines in *N*_1_, *N*_2_, and *J* with little substantial change in *N*_0_).

The impacts of invasive species on native species due to competition and resource use in the Mojave Desert are well recognized. A greenhouse experiment comparing *Bromus* spp. to native annuals found its root‐surface area and exploitation of deep soils allowed it to use water more rapidly than the natives, leading to greater biomass and N content as well as larger seeds that germinated at higher rates than native annual seeds (DeFalco, Bryla, Simth‐Longozo, & Nowak, 2003). Consistent with the greenhouse experiments, field experiments have reported thinning of invasive grasses results in an increase in total density and biomass of native herbaceous plants (Brooks, 2000). Our data give a strong indication that these relatively small‐scale experimental findings provide plausible mechanisms for the patterns we observed at community and ecoregional scales.

One implication of the overlapping distributions of the four invasive taxa is that effects of one species could substitute for those of another species that is either not present or present at low abundance. For example, *E*. *cicutarium* does not affect fuel loads or alter fire regimes in the Mojave Desert, even in very heavy rainfall years aboveground biomass dries by early to mid‐June and is easily blown away by strong winds. However, it can be a strong competitor (Kimball & Schiffman, 2003) and impact vegetation community structure and species composition following fire (Brooks & Matchett, 2003; Callison, Brotherson, & Bowns, 1985; Klinger & Brooks, 2017). Thus, in burned areas where cover of annual grasses is relatively low, increased productivity of *E*.* cicutarium* may still result in reduced cover and diversity of native herbaceous species through potential competitive interactions (Brooks, 2003). Forb cover in the Mojave Desert becomes greater with increasing time since fire and *E*. *cicutarium* cover often dominates the assemblage of forbs, so the magnitude of these effects could become more pronounced over time.

A perhaps even more important implication of the overlapping niches is the combined influence of the species (greater invasive cover due to joint positive responses to fire by two or more of the species) could drive ecosystem changes even if conditions are suboptimal for each. MacDougall and Turkington (2005) distinguished between invasive species whose abundance increases after disturbance but do not drive ecosystem change (“passenger” species) and those that increase in abundance and drive ecosystem changes (“driver” species). The degree to which a species is a passenger or a driver may depend on environmental conditions. For example, *Schismus* spp. and *B*. *tectorum* may be meaningfully classified as drivers of change in fire regimes at lower and higher elevations, respectively, in the Mojave Desert (Brooks & Berry, 2006; Brooks et al., 2018). These are their elevational optima, but their distributions extend much beyond the optima. It becomes less likely either species would individually drive changes as the distance from their optima increased, so if fire occurred in such areas they would probably be passenger species. But even if habitat conditions are suboptimal, they can still be suitable for species to persist. If they co‐occurred in this situation, the species could potentially contribute enough biomass to promote changes in the probability of ignition, extent of fire, or other components of the fire regime. In effect, their co‐occurrence could drive changes that might not otherwise happen if one or the other species was not present.

Invasion syndromes, which are defined as recurring patterns in the interactions between invasive species and their environment, have been proposed as a means to integrate findings from studies focused on invasive species (Kueffer, Py, & Richardson, 2013). One pattern that certainly falls into the concept of an invasion syndrome is the transformation of shrub‐dominated ecosystems in the deserts of North American to invasive‐dominated herbaceous ecosystems as a result of frequent fire. To date, the studies of this interaction have focused almost exclusively on species that would be classified as transformers (Richardson et al., 2000), such as cheatgrass and red brome (Bradley & Mustard, 2006; Brooks & Chambers, 2011). There is little doubt these species have had profound transformative effects on arid ecosystems, but most systems have multiple invaders present and there is increasing evidence of the importance of their cumulative effects (Kuebbing, Nuñez, & Simberloff, 2013). Our study indicated co‐occurring invasive species may extend the impacts beyond that of a single species, even if that species is often considered a transformer. This underscores the need to expand the framework of this arid system invasion syndrome so it adequately represents the potential and real impacts from multiple species.

### 4.1 Conservation implications

Understanding the potential and actual impacts of co‐occurring invasive species is particularly important in arid and semiarid ecosystems, given the often irreversible outcomes associated with invasive plants and large‐scale conversion of one type of desert plant community to another (Olsson, Betancourt, McClaran, & Marsh, 2012). This may be crucial for resource managers in the Mojave Desert, which has not undergone the extensive landscape transformations as some desert regions in North America have, such as the Great Basin.

In terms of practical application, our data are helping resource managers better predict the vulnerability of sites to wildfire and evaluate postfire impacts on native vegetation communities. Data from our SDMs have been integrated into spatial fire management plans, used to inform fire suppression and postfire mitigation actions, and help evaluate the potential for postfire dominance of invasive species in restoration sites. In addition, the SDM and plot data are being used to help prioritize monitoring and restoration activities to help ensure the persistence of native‐dominated communities and reduce their vulnerability to postfire transformations (Brooks & Klinger, 2009). Study outputs such as the integration of the potential distribution of more than one species can help guide management decision‐making. For example, although many areas of the Mojave Desert show high suitability to one or more invasives, there are also low suitability areas (Figure 5). These sites could offer opportunities for focusing restoration efforts. Applications such as these are not limited to the North American deserts, but are directly transferable to most systems.

## CONFLICT OF INTEREST

None declared.

## AUTHOR CONTRIBUTIONS

All of the authors designed the study, RCK conducted the statistical analyses of the field plot data, and ECU developed the species distribution models. ECU and RCK wrote the manuscript, with contributions from MLB.

## Data Availability

Data deposited at USGS (https://doi.org/10.5066/P9GUST4Q).
