# Supplementary material for: Effects of invasive plants on fire regimes and postfire vegetation diversity in an arid ecosystem
Source: Ecol Evol. 2019 Nov 4;9(22):12421–35. doi: 10.1002/ece3.5650 (PMC6875662; doi:10.1002/ece3.5650)
Supplement: Supplementary file 1 [file ECE3-9-12421-s001.docx]

**SUPPLEMENTARY MATERIAL**

| **Table S1.** The number of 0.1 ha plots for sampling herbaceous vegetation in three elevation zones (Low < 1200 m; Mid = 1200 m to 1700 m; High > 1700 m) in the Mojave Desert, USA. We sampled each plot once in either 2009, 2010, 2012, or 2013. YPF = the number of years post-fire (time since last known fire). | | | |
| --- | --- | --- | --- |
|  | **Elevation Zone** | | |
| **YPF** | **Low** | **Mid** | **High** |
| Unburned | 73 | 61 | 95 |
| 1 to 5 | 136 | 91 | 47 |
| 6 to 10 | 21 | 24 | 31 |
| 11 to 20 | 55 | 17 | 46 |
| 21 to 40 | 34 | 38 | 38 |

**Table S2**. Correlations between potential input variables run on 2684 records for all four invasive plant taxa

| **Correlations >=0.8** | **Omit** | **Rationale** |
| --- | --- | --- |
| DEM and mean, min, max temperature | DEM | Effects of DEM will be reflected in other variables such as NDVI |
| % bare ground and % herbaceous cover | % herbaceous | Assumed % bare ground mapped with higher accuracy from MODIS-derived data ([DiMiceli et al. 2011](#_ENREF_16)) |
| Annual mean precipitation and spring and winter precipitation | Spring and winter precipitation | Mean precipitation more meaningful |
| NDVI_StdDev and NDVI Peak | NDVI_StdDev | Assumed more informative than peak NDVI |
| Mean temperature and min temperature | Min temperature | Mean temperature more informative |
| Mean temperature and max temperature | Max temperature | Mean temperature more informative |

**Table S3.** The co-occurrence matrix of the four invasive plant taxa based on the 618 plots in which they were present

|  | **Brorub** | **Brotec** | **Erocic** | **Schbar** |
| --- | --- | --- | --- | --- |
| **Brorub** | **87.5** | 54.2 | 74.1 | 31.9 |
| **Brotec** | 335 | **60.2** | 47.4 | 15.9 |
| **Erocic** | 458 | 293 | **76.7** | 31.9 |
| **Schbar** | 197 | 98 | 197 | **33.9** |

| The main diagonal (in **bold)** is the percentage of plots each species occurred in |  |
| --- | --- |
| The lower off-diagonal cells are the number of plots a pair of species co-occurred in | |
| The upper off-diagonal cells are the percentage of plots a pair of species co-occurred in | |

**Table S4.** Classes used to categorize predicted suitability values ranging from 0-1 into four classes (very high, high, medium, and low) for ease of interpretation using Jenk’s natural breaks for *Erodium cicutarium* and two bromus species

|  | **Low** | **Med** | **High** | **Very high** |
| --- | --- | --- | --- | --- |
| *Erodium cicutarium* | 0-0.18 | 0.19-0.47 | 0.48-0.79 | 0.80-1 |
| 2 *Bromus* spp. combined | 0-0.38 | 0.39-0.64 | 0.65-0.85 | 0.85-1 |

**Model Selection**

*Relationship between cumulative cover and co-occurrence of the four invasive taxa*

The random slope + intercept model of the relationship between the co-occurrence of the four taxa and their cumulative cover had complete support (ΔAICc = 30.6, *w*AICc = 1).

*Relationship of absolute and relative cumulative cover of the four invasive taxa with fire frequency and the number of years post-fire*

There was complete support for the model of the relationship invasive absolute cover had with fire frequency and the non-linear effect of YPF (Table S5). Fire frequency and the non-linear effect of YPF also had the most support in the model set for the relationship with relative cover of the invasives, but the fire frequency model also had some support (AICc = 1.7, *w*AICc = 0.27. The 95% CIs of the YPF parameters did not overlap zero though (Table S5), so we selected the fire frequency + YPF^2^ model as the most appropriate.

*Relationship of the diversity and evenness indices with relative cumulative cover of the four invasive taxa*

There were similar levels of support for the random intercept and random intercept + slope models for N_0_, (ΔAICc = 0.97, *w*AICc = 0.62), so based on the principal of parsimony we selected the random intercept model. There was complete support for the model with the non-linear effect of invasive relative cover on N_0_ (Table S5).

There was complete support for the random intercept + slope models for N_1_ (ΔAICc = 180.3, *w*AICc = 1), N_2_ (ΔAICc = 164.9, *w*AICc = 1), and *J* (ΔAICc = 61.9, *w*AICc = 1). There was also complete support for the model with the non-linear effect of invasive relative cover on N_1_, N_2_, and *J* (Table S5).

| **Table S5.** Model selection statistics for: (A and B) the relationship of absolute and relative cover of four invasive plants taxa with the number of years post-fire (YPF) and fire frequency (Frequency, the number of times a plot burned since 1974); and, (C, D, E, and F) the relationship three indices of herbaceous plant diversity (N_0_, N_1_, N_2_) and an index of herbaceous plant evenness (*J*) had with relative cover of the four invasive plant taxa. | | | | | |
| --- | --- | --- | --- | --- | --- |
| **Model** | **K** | **AICc** | **ΔAICc** | ***w*AICc** | **Cumulative *w*AICc** |
| 1. ***Absolute cover*** |  |  |  |  |  |
| YPF^2^ + Frequency | 6 | 1993.6 | 0.0 | 1 | 1 |
| YPF + Frequency | 5 | 2014.8 | 21.1 | 0 | 1 |
| Frequency | 4 | 2016.3 | 22.7 | 0 | 1 |
| Null | 3 | 2074.8 | 81.2 | 0 | 1 |
| YPF | 4 | 2076.4 | 82.7 | 0 | 1 |
|  |  |  |  |  |  |
| 1. ***Relative cover*** |  |  |  |  |  |
| YPF^2^ + Frequency | 6 | 2801.5 | 0.0 | 0.63 | 0.63 |
| Frequency | 4 | 2803.2 | 1.7 | 0.27 | 0.90 |
| YPF + Frequency | 5 | 2805.2 | 3.7 | 0.10 | 1.0 |
| YPF | 4 | 2842.6 | 41.1 | 0.0 | 1.0 |
| Null | 3 | 2844.4 | 42.9 | 0.0 | 1.0 |
|  |  |  |  |  |  |
| 1. ***N*_0_** |  |  |  |  |  |
| Invasive Cover^2^ | 7 | 4460.0 | 0.0 | 1 | 1 |
| Invasive Cover | 6 | 4487.6 | 27.6 | 0 | 1 |
| Null | 5 | 4487.9 | 27.9 | 0 | 1 |
|  |  |  |  |  |  |
| 1. ***N*_1_** |  |  |  |  |  |
| Invasive Cover^2^ | 7 | 3238.7 | 0.0 | 1 | 1 |
| Invasive Cover | 6 | 3309.7 | 71.0 | 0 | 1 |
| Null | 5 | 3431.2 | 192.5 | 0 | 1 |
|  |  |  |  |  |  |
| 1. ***N*_2_** |  |  |  |  |  |
| Invasive Cover^2^ | 7 | 2898.1 | 0.0 | 1 | 1 |
| Invasive Cover | 6 | 2934.2 | 36.1 | 0 | 1 |
| Null | 5 | 3042.6 | 144.5 | 0 | 1 |
|  |  |  |  |  |  |
| 1. ***J*** |  |  |  |  |  |
| Invasive Cover^2^ | 7 | 1823.2 | 0.0 | 1 | 1 |
| Invasive Cover | 6 | 1951.3 | 128.0 | 0 | 1 |
| Null | 5 | 1993.3 | 170.0 | 0 | 1 |

**Figure S1**
