# Supplementary material for: Effects of invasive plants on fire regimes and postfire vegetation diversity in an arid ecosystem
Source: Ecol Evol. 2019 Nov 4;9(22):12421–35. doi: 10.1002/ece3.5650 (PMC6875662; doi:10.1002/ece3.5650)
Supplement: Supplementary file 2 [file ECE3-9-12421-s002.docx]

1 m x 1 m

**(A)**

3.2 m x 3.2 m

**IV**

**I**

**II**

**III**

32 m x 32 m

10 m x 10 m

16 m

**Figure S1.** Layout of 0.1 ha macroplots used in a study of post-fire succession patterns in the Mojave Desert. The figure shows: (A) the arrangement of nested subplots used for recording species richness of all vascular plants that occurred in the plots (subplot sizes = 1, 10, 100, and 1000 m²); and, (B) the randomly 1 m² quadrats used to estimate cover of all herbaceous species.

**(B)**

27 m

17 m

8 m

Figure S1 continued.
